# Changes in diabetes prevalence and corresponding risk factors - findings from 3- and 6-year follow-up of PURE Poland cohort study

**DOI:** 10.1186/s12889-020-08970-5

**Published:** 2020-06-03

**Authors:** Katarzyna Zatońska, Alicja Basiak-Rasała, Dorota Różańska, Maciej Karczewski, Maria Wołyniec, Andrzej Szuba, Katarzyna Połtyn-Zaradna

**Affiliations:** 1 Department of Social Medicine, Wroclaw Medical University, O. Bujwida 44 Street, 50-345 Wrocław, Poland; 2 Department of Dietetics, Wroclaw Medical University, ul. Parkowa 34, 51-616 Wrocław, Poland; 3 The Faculty of Environmental Engineering and Geodesy, Departament of Mathematics, Wroclaw University of Enviromental and Life Sciences, ul. Grunwaldzka 53, 50-357 Wrocław, Poland; 4 Department of Angiology, Hypertension and Diabetology, Wroclaw Medical University, ul. Borowska 213, 50-556 Wrocław, Poland

**Keywords:** Diabetes, PURE, IFG, Cohort, Longitudinal

## Abstract

**Background:**

Diabetes mellitus (DM) is one of the greatest challenges for public health worldwide. The aim of the study was the analysis of diabetes development in participants with normoglycemia and Impaired Fasting Glucose (IFG) in 3-year and 6-year follow-up of PURE Poland cohort study.

**Methods:**

The analysis was conducted in Polish cohort enrolled into Prospective Urban and Rural Epidemiology (PURE) Study. The following study presents results of 1330 participants that have partaken both in the baseline study, in 3-year and in the 6-year follow up. The analysis of the impact of risk factors on diabetes development was performed using multivariate Cox frailty analysis. Population Attributable Risk (PAR) was computed individually for every risk factor.

**Results:**

Diabetes prevalence increased from 17.7% at baseline to 23.98% in 3-year- and 28.27% in 6-year follow-up. The risk of diabetes was higher in participants with obesity [HR = 5.7, 95%Cl 2,56-12,82], overweight [HR = 3.4, 95%Cl 1,56-7,54] and IFG [HR = 2.7, 95%Cl 1,87-3,85]. The risk of diabetes development was almost 2-fold higher in men than in women [HR = 1.826; 95%CI =1,24 - 2,69]. In 6 years, diabetes developed in 23.8% of participants with IFG and 7.9% of participants with normoglycemia. According to PAR, overweight and obesity accounted for 80.8%, hypertension for 67.6% and IFG for 38.3% of diabetes cases in our population.

**Conclusions:**

Our study reveals alarming increase in prevalence of diabetes during 6 years of observation. In our population, most diabetes cases can be attributed to overweight, obesity, hypertension and IFG. Findings add strong rationale to implement targeted preventive measures in population of high risk.

## Background

Diabetes mellitus (DM) is one of the greatest challenges for public health worldwide. In 2016 DM was seventh leading cause of death [1]. The worldwide prevalence of diabetes has doubled from 4.7% in 1980 to 8.5% in 2014. International Diabetes Federation (IDF) estimates, that the global number of people with diabetes will rise from 425 million in 2017 to 629 million in 2045 [2]. Type 2 diabetes mellitus comprises the majority of diabetes cases, although its risk factors are largely modifiable. The interventions, which can prevent or delay onset of diabetes are maintaining healthy diet, improving physical activity, maintaining a normal body weight and avoiding tobacco smoking [1]. Impaired fasting glucose (IFG) and impaired glucose tolerance (IGT), which can precede the onset of diabetes become more and more prevalent. IFG and IGT are independent risk factors of cardiovascular diseases [3], presumably due to contribution to endothelial dysfunction [4], increased oxidative stress and inflammation in comparison to normoglycemic people [5]. It is estimated that diabetes is responsible for two-fold excessive risk for coronary heart disease, stroke and vascular-related deaths independently from other risk factors [6]. The same authors concluded that 10% of deaths related to cardiovascular conditions in developed countries can be attributed to diabetes. Causal link between diabetes and macrovascular complications (coronary heart disease, stroke) and microvascular complications (neuropathy, nephropathy, retinopathy) has been clearly established, but more evidence emerge on the link between diabetes and conditions like cancer, dementia, liver diseases [7]. 8% of the Polish population has diabetes, and it is estimated that in 2040 the number will increase to 11% of the population. It is estimated, that 25–30% of adults with diabetes in Poland are unaware of their condition [8].

Data regarding longitudinal observation of diabetes prevalence in Poland is limited. The analysis from cross-sectional study NATPOL 2002 to NATPOL 2011, revealed, that prevalence of diabetes over the years was rather stable (6.7% in 2002 and 6.8% in 2011) [9]. On the other hand, comparison of diabetes prevalence in randomized Multi-center National Population Health Survey (WOBASZ) I (2003–2005) and II (2013–2014) indicated than over 10-year period, the prevalence of diabetes increased from 6.8 to 9.8% of the population [10]. In the first stage of the study, WOBASZ I, Lower Silesia province had one of the lowest prevalence of diabetes among other provinces in Poland – 5.8% in men and 5.9% in women [11]. Information from database of Polish National Health Fund (NFZ) indicated that prevalence of diabetes in Lower Silesia slightly increased from 5.8 persons per 100,000 inhabitants in 2008 to 6.6 persons per 100,000 inhabitants in 2013 [12].

Poland was one of 21 countries enrolled in Prospective Urban and Rural Epidemiology Study (PURE). Prospective design of the study provided an opportunity to observe the cohort over several years. The first stage of the cohort study was conducted between 2007 and 2010 and participants were enrolled from both urban and rural areas from Lower Silesia province in Poland. The aim of the study was the analysis of diabetes development in participants with normoglycemia and IFG during 6 years of observation of PURE Poland study cohort. The analysis will shed light on diabetes prevalence over the years and corresponding risk factors.

## Methods

**Fig. 1:**
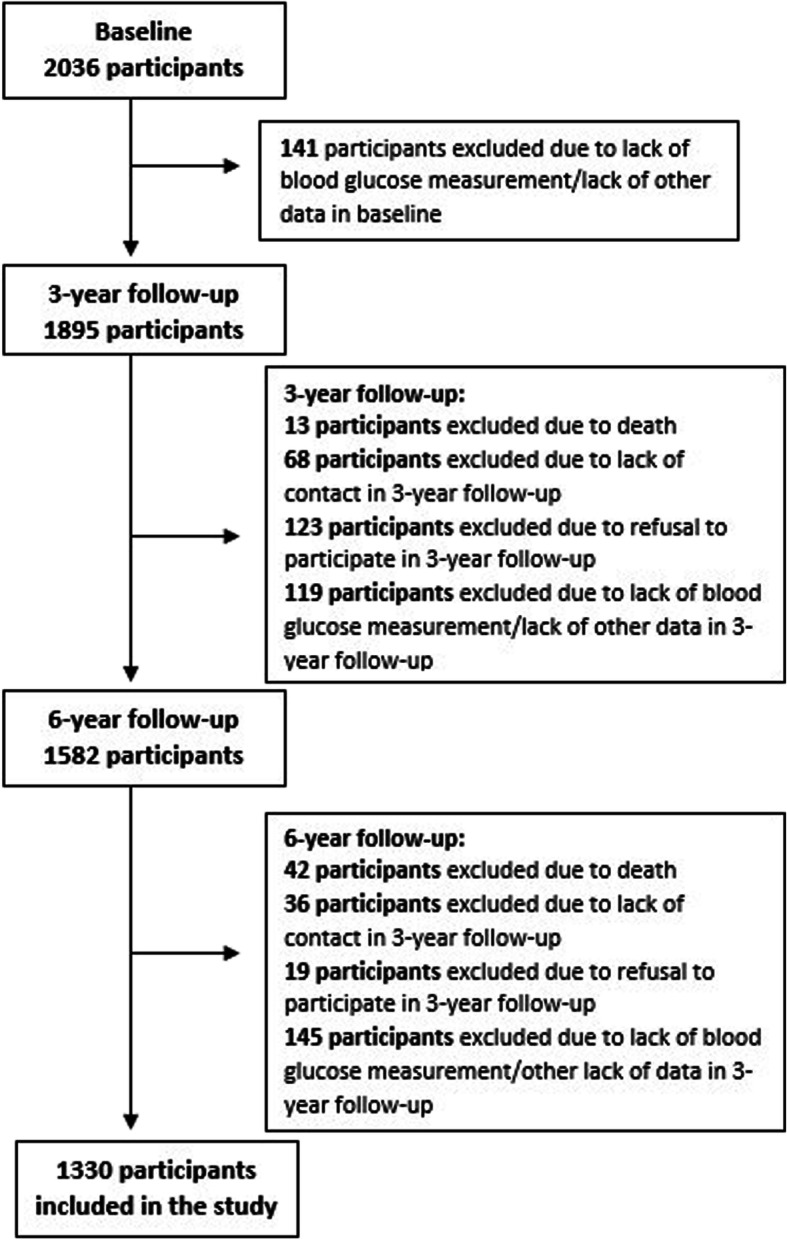
Study group selection

In our cohort we followed protocol of global PURE study [13, 14]. PURE is an international study comprising cohorts from 21 high-, middle- and low income countries, aiming to observe biological, socioeconomic, behavioral and environmental factors of mortality and morbidity of noncommunicable diseases [13]. Study protocol included questionnaire study (individual health questionnaire, family questionnaire, food frequency questionnaire, all developed specially for the PURE study and international physical activity IPAQ questionnaire [15]), anthropometric measurements, blood pressure measurement, ECG, spirometry, blood and urine collection [14]. Follow-ups are conducted with consistent protocol every 3 years. In Polish cohort, participants were enrolled and assessed at baseline between 2007 and 2010. The baseline cohort consisted of 2036 adult participants (1277 women), 59.4% from urban and 40.6% from rural area (Lower Silesia province, Poland). The enrolled population was 30–85 years old. Selection of the study group for hereby analysis is presented in Fig. 1. The hereby study was conducted in 1330 individuals, who participated both at the baseline study, in 3-year and in the 6-year follow up and whose data regarding measurement of fasting glucose and diabetes history was complete at every stage of the study. To the normoglycemia group comprised participants with fasting plasma glucose between 70 and 99 mg/dL (3,9–5.5 mmol/L) and who didn’t self-report diabetes diagnosed in the past. IFG group comprised individuals, whose fasting plasma glucose ranged between 100 mg/dL (5.6 mmol/L) and 125 mg/dL (6.9 mmol/L). Diabetes group comprised individuals, whose fasting plasma glucose was 126 mg/dL (7.0 mmol/L) or higher, or those, who self-reported diabetes diagnosis and diabetic treatment. Assessment of the glucose metabolism, expressed as classification to three mentioned groups: normoglycemia, IFG and diabetes, was performed at three stages of the study: at baseline, 3-year and 6-year follow-up. The aim of the study was the analysis of diabetes development with corresponding risk factors, in participants with normoglycemia and IFG during 6 years of observation of PURE Poland study cohort. Body mass was assessed with the use of Body Mass Index (BMI), whereas occurrence of abdominal obesity was assessed with the use of Waist-to-Hip Ratio (WHR). Participants were divided into four groups considering their BMI values: underweight (BMI < 18,5 kg/m^2^), normal body weight (BMI 18,5 -24,99 kg/m^2^), overweight (BMI 25,00–29,99 kg/m^2^) and obesity (BMI ≥30,00 kg/m^2^). Abdominal obesity on the basis of WHR was determined, when WHR in men was ≥0,94 and in women ≥0,80. To account for different time of developing diabetes, analysis of the impact of risk factors was performed using multivariate Cox frailty analysis. The strength of the effect was described using hazard ratios (HR) with 95% confidence intervals. The multivariable model was adjusted to variables included age, sex, urban or rural location, education, marital status, BMI, waist-to-hip ratio, smoking status, hypertension and use of statins, and used age and sex as random effects. All sociodemographic variables, BMI, WHR, hypertension and use of statins, that we refer to in the model were recorded at the baseline. The analysis was performed in R for Windows (version 3.5.3) [16]. Population Attributable Risk (PAR) was computed according to Crowson et al. [17] individually for every risk factor. In order to calculate PAR some categories of the risk factors were adjusted to dichotomous variables: overweight + obesity vs. normal body weight; age < 45 years old vs. age > 45 years old; marital status: in a relationship (married+in a relationship) vs. not in a relationship (divorced+never married+widowed+single); education: primary education+trade school vs. secondary+higher education; smoking status: current smoker vs. never smoker+ex-smoker. Written and informed consent was gathered from all individuals taking part in the study. The study has been reviewed and accepted by The *Bioethics Committee* of the *Wrocław Medical University (positive opinion nr KB-443/2006).*

## Results

Sociodemographic characteristic of baseline cohort is presented in Table 1. At the baseline 17.7% of participants had diabetes, 25.9% had IFG and 56.4% were normoglycemic. Diabetes was statistically significantly more prevalent in men than women. At baseline, the number of men with diabetes was 5% higher, than women with diabetes (20.8% vs. 15.8% respectively). At this stage of the study proportions of participants with IFG and normoglycemia in men and women were comparable: in IFG group, 24.2% vs. 26.9% respectively and in normoglycemic group 55.0% vs. 57.2% respectively. Diabetes was significantly more prevalent in participants living in rural than in urban areas (25.9% vs. 12.7% at baseline) (Table 2).

**Table 1:** Baseline characteristics of 1330 participants of PURE Poland cohort study

	Total	Normoglycemia	IFG	Diabetes
**Overall***n(%)*	1330 (100)	750 (56.39)	345 (25.94)	235 (17,67)
*Sex*				
**Women***n(%)*	839 (63.08)	480 (57.21)	226 (26.94)	133 (15.85)
**Men***n(%)*	491 (36.92)	270 (54.99)	119 (24.24)	102 (20.77)
**Age**, *mean years ± sd*	54.42 ± 9.39	52.95 ± 9.25	54.9 ± 9.12	58.40 ± 9.06
*Place of residence*				
**Urban***n(%)*	828 (62.26)	537 (64.86)	186 (22.46)	105 (12.68)
**Rural***n(%)*	502 (37.74)	213 (42.43)	159 (31.67)	130 (25.90)
*Education*				
**Primary***n(%)*	178 (13.38)	71 (39.89)	45 (25.28)	62 (34.83)
**Secondar**y *n(%)*	524 (39.40)	292 (55.73)	144 (27.48)	88 (16.79)
**Trade school***n(%)*	194 (14.59)	97 (50.0)	55 (28.35)	42 (21.65)
**University***n(%)*	434 (32.63)	290 (66.82)	101 (23.27)	43 (9.91)
*Marital status*				
**Married***n(%)*	1009 (75.92)	584 (43.94)	252 (18.96)	173 (13.02)
**Divorced***n(%)*	99 (7.45)	48 (3.6)	35 (2.6)	16 (1.2)
**Never married***n(%)*	81 (6.1)	47 (3.5)	21 (1.6)	13 (0.97)
**Widowed***n(%)*	140 (10.53)	71 (5.34)	37 (2.7)	32 (2.4)
**BMI**, mean ± sd kg/m2	28.17 ± 5.19	26.99 ± 4.52	28.78 ± 5.15	31.02 ± 5.94
**WHR Women***mean ± sd*	0.841 ± 0.078	0.82 ± 0.068	0.854 ± 0.08	0.893 ± 0.08
**WHR Men***mean ± sd*	0.959 ± 0.066	0.944 ± 0.069	0.967 ± 0.072	0.993 ± 0.083
**WHR Women** abdominal obesity *n(%)*	554 (66.03)	278 (50.18)	161 (29.06)	115 (20.76)
**WHR Men** abdominal obesity *n(%)*	297 (60.49)	144 (48.49)	73 (24.58)	80 (26.93)
**Current smokers***n(%)*	246 (18.5)	137 (55.69)	67 (27.24)	42 (17.07)
**Former smokers***n(%)*	433 (32.56)	232 (53.58)	114 (26.33)	87 (20.09)
**Never smokers***n(%)*	651 (48.95)	381 (58.53)	164 (25.19)	106 (25.19)
**Statins** n(%)	163 (12.26)	78 (47.85)	37 (22.70)	48 (29.45)
**Hypertension** n(%)	891 (66.99)	442 (49.61)	246 (27.61)	203 (22.78)

*Abbreviations: IGF* Impaired Fasting Glucose, *BMI* Body Mass Index, *WHR* Waist-to-hip ratio

**Table 2:** Prevalence of diabetes, IFG and normoglycemia in PURE Poland Study population in baseline, 3-year and 6-year follow-up

	Baseline	*p*-value	Follow-up 3	*p*-value	Follow-up 6	*p*-value
Diabetes prevalence						
Total	235 (17,67%)		319 (23,98%)		376 (28,27%)	
Female	133 (15,85%)	0.02	175 (20,86%)	< 0.01	204 (24,31%)	< 0.01
Male	102 (20,77%)		144 (29,33%)		172 (35,03%)	
Urban	105 (12,68%)	< 0.01	150 (18,12%)	< 0.01	182 (21,98%)	< 0.01
Rural	130 (25,9%)		169 (33,67%)		194 (38,65%)	
IFG prevalence						
Total	345 (25.94%)		288 (21.65%)		229 (17.22%)	
Female	226 (26.94%)	> 0.05	157 (18.71%)	< 0.01	141 (16.81%)	> 0.05
Male	119 (24.24%)		131 (26.68%)		88 (17.92%)	
Urban	186 (22.46%)	< 0.01	121 (14.61%)	< 0.01	124 (14.98%)	< 0.01
Rural	159 (31.67%)		167 (33.07%)		105 (20.92%)	
Normoglycemia prevalence						
Total	750 (56.39%)		723 (54.36%)		725 (54.51%)	
Female	480 (57.21%)	> 0.05	507 (60.43%)	< 0.01	494 (58.88%)	< 0.01
Male	270 (54.99%)		216 (43.99%)		231 (47.05%)	
Urban	537 (64.86%)	< 0.01	557 (67.27%)	< 0.01	522 (63.04%)	< 0.01
Rural	213 (42.43%)		166 (33.07%)		203 (40.44%)	

We observed higher increase in diabetes prevalence between baseline and 3-year follow-up (from 17.7 to 23.9%), than between 3-year and 6-year follow-up (from 23.9 to 28.3%). In 6-year follow-up there was 1.5-fold increase in diabetes’ prevalence (17.7% in baseline vs. 28.3% in 6-years follow-up). The prevalence of IFG and normoglycemia decreased to 17.2 and 54.5% respectively (Table 2). Between baseline and 6-year follow-up, diabetes developed in 23.8% of participants with IFG and 7.9% of participants with normoglycemia. In 6-years follow-up, the prevalence of diabetes in men increased 1.7-fold to reach 35.0%. Slightly lower, 1.5-fold increase was observed in women (24.3%) (Table 2). In 6-year follow-up, number of participants with IFG decreased both in men (17.9%) and in women (16.8%). In the normoglycemic group, we observed slight increase in women (58.9%) and statistically significant decrease in men (47.0%). Among men with IFG diabetes developed in 35.3% of participants, and in women in 17.7%. Among men with normoglycemia, diabetes developed in 10.4%, and in women in 6.4%. In all analyzed periods, the prevalence of diabetes was higher among men than women. The risk of diabetes development was almost 2-fold higher than in women [HR = 1826; CI =1,24 - 2,69] (Table 4). In both analyzed periods of time, diabetes developed statistically significantly more often in participants with IFG, than in participants with normoglycemia (Table 3).

**Table 3:** Changes in glycemic status in 6 years of observation (“0” – Baseline, “3” – 3-year follow-up, “6” – 6-year follow-up)

Total n (%)			
	to Normoglycemia	to IFG	to Diabetes
Study period			
0–3			
from normoglycemia	551 (73,47%)	167 (22,27%)	32 (4,27%)
from IFG	172 (49,86%)	121 (35,07%)	52 (15,07%)
0–6			
from normoglycemia	563 (75,07%)	128 (17,07%)	59 (7,87%)
from IFG	162 (46,96%)	101 (29,28%)	82 (23,77%)
3–6			
from normoglycemia	614 (84,92%)	91 (12,59%)	18 (2,49%)
from IFG	111 (38,54%)	138 (47,92%)	39 (13,54%)
Women			
0–3			
from normoglycemia	377 (78,54%)	87 (18,12%)	16 (3,33%)
from IFG	130 (57,52%)	70 (30,97%)	26 (11,50%)
0–6			
from normoglycemia	379 (78,96%)	70 (14,58%)	31 (6,46%)
from IFG	115 (50,88%)	71 (31,42%)	40 (17,69%)
3–6			
from normoglycemia	429 (84,62%)	65 (12,82%)	13 (2,56%)
from IFG	65 (41,40%)	76 (48,41%)	16 (10,19%)
Men			
0–3			
from normoglycemia	174 (64,44%)	80 (29,63%)	16 (5,93%)
from IFG	42 (35,29%)	51 (42,86%)	26 (21,85%)
0–6			
from normoglycemia	184 (68,15%)	58 (21,48%)	28 (10,37%)
from IFG	47 (39,50%)	30 (25,21%)	42 (35,29%)
3–6			
from normoglycemia	185 (85,65%)	26 (12,04%)	5 (2,32%)
from IFG	46 (35,11%)	62 (47,33%)	23 (17,56%)
Urban			
0–3			
from normoglycemia	438 (81,56%)	78 (14,53%)	21 (3,91%)
from IFG	119 (63,98%)	43 (23,12%)	24 (12,90%)
0–6			
from normoglycemia	423 (78,77%)	76 (14,15%)	38 (7,08%)
from IFG	99 (53,23%)	48 (25,81%)	39 (20,97%)
3–6			
from normoglycemia	480 (86,18%)	66 (11,85%)	11 (1,97%)
from IFG	42 (34,71%)	58 (47,93%)	21 (17,35%)
Rural			
0–3			
from normoglycemia	113 (53,05%)	89 (41,78%)	11 (5,16%)
from IFG	53 (33,33%)	78 (49,06%)	28 (17,61%)
0–6			
from normoglycemia	140 (65,73%)	52 (24,41%)	21 (9,86%)
from IFG	63 (39,62%)	53 (33,33%)	43 (27,04%)
3–6			
from normoglycemia	134 (80,72%)	25 (15,06%)	7 (4,22%)
from IFG	69 (41,32%)	80 (47,90%)	18 (10,78%)

In our cohort, IFG was found a significant risk factor of diabetes development. The risk of diabetes in participants with IFG was 2.7-fold higher than in participants with normoglycemia (Table 4). In our study, factors, which statistically differentiated diabetes prevalence were sex and place of residence.

**Table 4:** Risk of diabetes development according to selected risk factors (hazard ratios (HR) with 95% of confidence interval (CI)

Variable	HR (95% CI)
IFG	2.685 (1,87 - 3,85)^a^
Sex: Male	1.826 (1,24 - 2,69)^a^
Age > 64	3.175 (1,27 - 7,94)^a^
Age 45–64	2.634 (1,18 - 5,89)^a^
Urban place of residence	1.042 (0,68 - 1,59)
Education: College/University	0,555 (0,28 - 1,09)
Education: Secondary	1.081 (0,61 - 1,93)
Education: Trade School	1.084 (0,57 - 2,04)
Marital Status: Divorced	1.153 (0,58 - 2,31)
Marital Status: Never married	0,639 (0,23 - 1,78)
Marital Status: Widowed	1.047 (0,59 - 1,87)
BMI: Obesity	5.731 (2,56 - 12,82)^a^
BMI: Overweight	3.428 (1,56 - 7,54)^a^
WHR: Abdominal obesity	1.625 (0,96 - 2,74)
Statins	1.027 (0,64 - 1,65)
Currently Uses Tobacco Products	1.195 (0,71 - 1,99)
Formerly Used Tobacco Products	1.412 (0,95 - 2,10)
Hypertension	1,83 (1,01 - 3,31)^a^

^a^statistically significant results

In all analyzed periods, the proportion of participants with diabetes in rural areas was 2-fold higher than in urban areas. Other factors, which significantly increased the risk of diabetes in our population were: age, obesity and hypertension. After reaching 45 years of age, the risk of diabetes development increased 2.6-fold in age group 45–64 years and 3.2-fold in age group above 64 years. Obesity, ascertained on the basis of BMI, increased the risk of diabetes 5.7-fold and hypertension almost 2-fold (Table 4).

Percent PARs of the factors associated with diabetes risk are presented in Table 5. Overweight and obesity accounted for 80.8%, hypertension for 67.6% and IFG for 38.3% of diabetes cases in our population. Abdominal obesity ascertained on the WHR accounted for 61.3% of diabetes cases. The risk of diabetes development during this study period was lower in urban place of residence and among participants with higher education.

**Table 5:** Percent Population Attributable Risk (PAR) (with 95% confidence interval [CI]) of diabetes development according to selected risk factors

Variable	PAR (%)	PAR lower	PAR upper
IFG	38,31	26,09	48,51
Sex: Male	22,45	9,59	33,48
Age > 45	69,61	41,33	84,26
Urban place of residence	-34,74	−59,12	−14,09
Secondary+Higher education	−46,74	−79,56	−19,93
Marital status: Relationship	−1,46	−10,24	6,62
BMI: overweight+obesity	80,78	64,19	89,68
WHR: abdominal obesity	61,3	43,89	73,31
Statin	6,36	−0,04	12,35
Smoking: Current	−0,39	7,97	6,65
Hypertension	67,64	50,07	79,03

## Discussion

Our paper presents the results of one of a few longitudinal cohort studies in Poland. According to our findings, the prevalence of diabetes increased immensely over 6-years period of observation. The number of participants affected by diabetes increased 1.5-fold over the time period, even despite the fact, that baseline prevalence of diabetes was already quite high in our cohort. According to up-to-date, countrywide reports, 8% of Polish population has diabetes and it is expected that the prevalence will increase to 11% in 2040 [8]. Multi-center National Population Health Survey (WOBASZ) estimated prevalence of diabetes on 9.8% in 2014 [10]. In our cohort, 17.7% of participants had diabetes at the baseline and its prevalence progressed to 28.3% after 6 years. High baseline prevalence of diabetes in our cohort can be partially explained by higher percentage of older participants and higher prevalence of overweight and obesity than in general Polish population. In our cohort study, risk factors statistically significantly associated with diabetes progression were: increased Body Mass Index (BMI), IFG, male sex, increasing age, and hypertension. The analysis suggested, that in our population, intervention targeting predominantly excessive body mass, hypertension and IFG could result in significant decrease of diabetes risk.

In our study, the strongest risk factors of progression to diabetes were overweight and obesity, which is consistent with previously published studies [18, 19]. Conforming to our results, in Copenhagen General Population Study (CGPS) comprising 95,540 participants observed for median 4.7 years, the risk of type 2 diabetes increased stepwise along with the increase in BMI [20]. In CGPS study overweight increased the risk of type 2 diabetes 2.1-fold (95% Cl 1.7–2.5), whereas obesity increased the risk 6.4-fold (95% Cl 5.5–7.5) [20]. We observed similar values in our study (3.4-fold and 5.7-fold increase in diabetes risk respectively). According to Diabetes Prevention Program (DPP) Research Group, lifestyle intervention, aiming predominantly body mass reduction, was associated with 4-years delay in progression to diabetes in comparison to placebo, whereas metformin intake was associated with 2-years delay [19]. Furthermore, according to longitudinal analysis of Ligthart et al., obesity reduced the time lived with normoglycemia [18]. Our findings confirm, that reduction of excessive body mass is one of the most important lifestyle interventions in prevention of type 2 diabetes. Overweight and obesity accounted for 80.8% of diabetes cases in our population. Our observation are conforming to Hu et al., who also identified overweight and obesity as the most important determinants of diabetes development in longitudinal analysis of Nurses’ Health Study population [21]. According to PAR calculations in our population, overweight and obesity ascertained on the basis of BMI accounted for more diabetes cases than abdominal obesity ascertained on the basis of WHR (80.8% vs 61.3% respectively). Similarly, in longitudinal study by Nemesure et al., after 9 years of observation, higher WHR was related to higher risk of hypertension, but diabetes was more associated with increased BMI [22]. Having said that, lack of statistical significance between WHR and diabetes development in our cohort might have resulted from high prevalence of visceral adiposity according to WHR in analyzed population. Majority (approx. 60%) of our population had visceral adiposity according to applied criteria and abdominal obesity was also very prevalent in normoglycemic participants.

In our study, the risk of diabetes in participants with IFG was 2.7-fold higher than in participants with normoglycemia. This observation was consistent with results obtained in longitudinal analysis of Rotterdam Study, where it was estimated, that 75% of people with prediabetes at the age of 45 years, will develop full-symptomatic diabetes [18]. Similar estimates can be found in expert opinion of American Diabetes Association (ADA) [23]. Our findings confirm the current guidelines of Diabetes Poland, which emphasize, that participants with prediabetes should receive intensive lifestyle counselling, targeting predominantly improving physical activity and reducing body mass in order to prevent diabetes development [24]. It seems to be reasonable to target overweight and IFG as the most important risk factors. Early intervention in prediabetic patients, including improving diet and physical activity can delay or even cease progression to diabetes [25, 26]. In our cohort during 6 years of observation, more participants with IFG in baseline returned to normoglycemia, than progressed to diabetes (46.96% vs. 23.77% respectively). Although our study is an observational one, we cannot rule out, that participants introduced some lifestyle changes during this period of time which influenced their glycemic status. The beneficial impact of changes in lifestyle and following improvement of glucose metabolism in patients with IFG was previously described [25, 26].

In our cohort, diabetes was much more prevalent in men than women and its prevalence increased more rapidly in men over 6-years of observation. This observation is in line with other Polish studies [10, 11] and international reports [2]. As stated in NCD Risk Factor Collaboration report, higher prevalence of diabetes in men might be due to higher prevalence of other risk factors, like tobacco smoking and visceral adiposity [27]. According to Nordström et al., despite comparable BMI between men and women, larger amount of visceral fat in men was associated with higher prevalence of type 2 diabetes [28].

Although place of residence was not statistically significant risk factor of diabetes development in our model, high increase in prevalence of diabetes in rural population should be taken into consideration. Both in urban and rural populations the prevalence of diabetes increased during the course of the study, but in rural population the increase was 4% higher than in urban population. Higher prevalence of diabetes and other noncommunicable diseases in rural populations have been observed in other studies [29, 30]. Higher burden of diabetes in rural population of our cohort might be due to higher prevalence of risk factors like obesity, tobacco smoking and poor diet, than in urban population [31, 32]. On the other hand, as reported by IDF Diabetes Atlas, there are currently more people with diabetes in urban than rural areas globally [2]. That being said, the authors conclude that in mostly rural, low income countries, the proportion of undiagnosed diabetes could be as high as 69.2%, which might have contributed to underestimation of diabetes prevalence in rural place of residence [2]. In our findings, hypertension increased the risk of diabetes almost 2-fold. This association could be explained by common risk factors for hypertension and diabetes. On the other hand, there are longitudinal studies, in which hypertension was found an independent risk factor of type 2 diabetes. According to findings from The Korean Genome and Epidemiology Study, hypertension and prehypertension were associated with increased risk of type 2 diabetes independently from other factors, like BMI, sex and baseline glucose status [33]. Similarly to our findings, Conen et al., who analyzed data from the Women’s Health Study, observed that hypertension was responsible for 2-fold increase in the risk of diabetes over 10 years of observation [34]. Moreover, in the same study, the high normal blood pressure (130–139/85–89 mmHg) was associated with 1.5-fold increase in diabetes risk [34]. In our study, hypertension was also second most important population risk factor of diabetes according to PAR. Hypertension may often coexist with obesity, as a component of Cardiometabolic Syndrome (CardMets) or be triggered by excessive body weight [35]. In baseline, prevalence of hypertension was already higher than in general Polish population (60.3% of the participants were hypertensive [36]), which partially explain, why hypertension play an important role in modulating risk of diabetes in our population.

There are few limitations to consider in our study. PURE Poland was a cohort study and therefore the results should be treated with caution. Our cohort was characterized by overrepresentation of older participants as well as higher prevalence of obesity than general Polish population, which can partially explain high prevalence of diabetes in baseline. Prevalence of diabetes was self-reported and ascertained on the basis of fasting glucose level, but no glucose tolerance tests were performed, which could have caused some underestimation. On the other hand, the same methodology is used in global PURE study. There are also several strengths to consider. This is one of a few longitudinal cohort studies in Poland on a large sample of population, conducted with the same protocol during the course of the study.

## Conclusion

Our study reveals alarming increase in prevalence of diabetes during 6 years of observation. Overweight and obesity were one of the most important risk factors of progression to diabetes in PURE Poland cohort. In our population, most diabetes cases can be attributed to overweight, obesity, hypertension and IFG. Findings add strong rationale to implement targeted preventive measures in population of high risk.

## Data Availability

The datasets used and/or analyzed during the current study are available from the corresponding author on reasonable request.

## Abbreviations

PURE: Prospective Urban and Rural Epidemiology Study
PAR: Population Attributable Risk
IFG: Impaired fasting glucose
IGT: Impaired glucose tolerance
DM: Diabetes mellitus
IDF: International Diabetes Federation
WOBASZ: Multi-center National Population Health Survey
NFZ: Polish National Health Fund
BMI: Body Mass Index
WHR: Waist-to-Hip Ratio
HR: Hazard ratio
DPP: Diabetes Prevention Program
ADA: American Diabetes Association
